# Malaria-diabetes comorbidity is linked to higher parasitaemia and enhanced IgG response to malaria vaccine candidate antigens

**DOI:** 10.1371/journal.pone.0341659

**Published:** 2026-02-06

**Authors:** Gideon Agyare, David Courtin, Samuel Asamoah Sakyi, Gideon Kwesi Nakotey, Naa Adjeley Frempong, Prince Amoah Barnie, Samuel Kofi Tchum, Samuel Victor Nuvor, Benjamin Amoani

**Affiliations:** 1 Department of Microbiology and Immunology, School of Medical Sciences, University of Cape Coast, Cape Coast, Ghana; 2 UMR 261 MERIT, Université de Paris, Institute de Recherche pour le Développement (IRD), Paris, France; 3 Department of Molecular Medicine, School of Medical Sciences, Kwame Nkrumah University of Science and Technology, Kumasi, Ghana; 4 Department of Medical Laboratory Technology, Central College of Science and Technology, Agona Swedru, Ghana; 5 Department of Parasitology, Noguchi Memorial Institute for Medical Research, University of Ghana, Accra, Ghana; 6 Department of Forensic Science, School of Biological Sciences, College of Agriculture and Natural Sciences, University of Cape Coast, Cape Coast, Ghana; 7 Kintampo Health Research Centre, Ghana Health Service, Kintampo, Ghana; 8 Department of Biomedical Science, School of Allied Health Sciences, University of Cape Coast, Cape Coast, Ghana; Shanghai Jiao Tong University School of Medicine, CHINA

## Abstract

**Background:**

The coexistence of type 2 diabetes mellitus (T2DM) and malaria presents a significant public health concern, particularly in malaria-endemic regions. T2DM’s immunomodulatory effects may influence immune responses to infectious diseases, but its impact on naturally acquired antibodies against malaria vaccine candidate antigens remains unclear. This study investigated how T2DM-malaria comorbidity affects IgG responses to malaria vaccine candidate antigens (GLURP-R2, GLURP-RO, MSP3, MSP1, AMA-1 and CSP) among individuals in the Central Region of Ghana.

**Methods:**

This hospital-based case-control study recruited a total of 144 participants 40 with diabetes, 25 with both diabetes and malaria, 41 with malaria only, and 38 controls (hospital staff without malaria or diabetes matched by age and sex). Malaria status and parasitaemia were confirmed using microscopy, blood glucose levels were measured via glucometer, and antibody levels were quantified using indirect ELISA. Data were analyzed using parametric and non-parametric statistical methods.

**Results:**

Patients with malaria-diabetes comorbidity exhibited significantly higher parasitaemia levels compared to those with malaria alone [1702 (IQR1 = 926.50, IQR3 = 4102) vs. 932 (IQR1 = 722.50, IQR3 = 1321), p = 0.02]. Relative to the negative control group, IgG responses to GLURP-R2 were markedly elevated, showing a 1.60-fold increase in the comorbidity group (β = 0.47 [95% CI: 0.10–0.83], p = 0.01) and a 1.43-fold increase in the malaria-only group (β = 0.36 [95% CI: 0.04–0.69], p = 0.03). Among individuals with comorbidity, IgG levels to GLURP-R0 and MSP1 were also 1.43-fold higher (β = 0.36 [95% CI: 0.03–0.68], p = 0.03) and 1.42-fold (β = 0.35 [95% CI: 0.09–0.61], p < 0.05), respectively. Conversely, antibody responses to MSP3, AMA1, and CSP did not differ significantly between the study groups (p > 0.05). In the multivariate regression model adjusted for age and sex, individuals with comorbidity exhibited significantly elevated IgG responses, showing a 1.38-fold increase to GLURP-R0 (β = 0.32 [95% CI: 0.07–0.59], p = 0.027) and a 1.34-fold higher response to MSP1 (β = 0.29 [95% CI: 0.02–0.47], p = 0.048), relative to the malaria-only group.

**Conclusion:**

These findings suggest that diabetes may enhance malaria parasite multiplication while also augmenting IgG antibody responses to malaria vaccine candidate antigens in individuals with comorbidity. Further research is required to elucidate the mechanisms by which diabetes influences antibody responses to malaria infection and its potential implications for malaria vaccine development.

## Introduction

Type 2 diabetes mellitus (T2DM) and malaria continue to pose significant global health challenges [1]. While T2DM is a widespread concern across both developed and developing nations, malaria remains disproportionately concentrated in low-income regions, particularly in sub-Saharan Africa [2,3]. In 2021 alone, there were an estimated 247 million malaria cases worldwide, with the majority occurring in African countries, highlighting the disease’s persistent burden in tropical and subtropical regions [4]. The Central Region of Ghana has a malaria disease burden of 20.3% [5]. Despite advancements in vaccine development, vector control, and chemoprevention, achieving long-lasting immunity against malaria parasitaemia remains a challenge due to the parasite’s complex biology, genetic variability, and ability to evade the immune system [6,7].

However, T2DM is a chronic metabolic disorder characterized by persistent hyperglycemia resulting from insulin resistance, impaired insulin secretion, or both [8,9]. A combination of genetic predisposition and environmental factors, such as obesity, poor diet, and physical inactivity, contributes to the disease’s pathophysiology [10,11]. As of 2021, an estimated 537 million people globally were living with diabetes, with T2DM accounting for approximately 90% of cases [12,13]. In Ghana, urban populations have reported a T2DM prevalence of at least 6%, with incidence increasing with age and obesity [14,15].

Recent studies have highlighted the intricate relationship between T2DM and malaria, particularly in malaria-endemic regions [1,16,17]. A case-control study in Ghana reported that individuals with T2DM had a 46% higher risk of contracting *Plasmodium falciparum* infection [18]. Hyperglycemia in T2DM has been shown to enhance *P. falciparum* virulence by promoting parasite replication and rosette formation [19]. Additionally, experimental studies suggest that malaria infection itself may contribute to insulin resistance, potentially increasing the risk of T2DM in endemic regions [20].

The co-occurrence of T2DM and malaria significantly alters immune responses, leading to macrophage dysfunction, impaired natural killer cell activity, complement system abnormalities, and defects in humoral immunity [21–23]. Tumor necrosis factor-alpha (TNF-α), a key inflammatory mediator, plays a crucial role in both diseases, potentially linking their pathophysiologies through chronic inflammation [24,25]. However, our previous study found that increasing metformin dosage suppresses TNF-α levels [26]. This could influence the immune response to the malaria parasite in individuals with malaria-diabetes comorbidity [26]. Notably, protection against clinical malaria has been associated with cytophilic IgG subclasses, particularly IgG1 and IgG3 [27–29]. Higher antibody levels targeting merozoite surface proteins (MSP1, MSP2) and other malaria antigens such as AMA1, CSP, and GLURP have been correlated with reduced malaria incidence [30,31].

Understanding how metabolic disorders influence immune responses and the efficacy of vaccine candidate antigens in malaria-endemic regions is critical. This study aims to investigate the impact of T2DM-malaria comorbidity on IgG responses to *Plasmodium falciparum* (the most clinically important species globally [32]) malaria vaccine candidate antigens, contributing to the broader understanding of immune modulation in individuals with dual disease burdens.

## Methodology

### Study design and site

This case-control study was conducted in two distinct locations in Ghana: namely, the Cape Coast Metropolis (CCM) and Agona West Municipality (AWM). Rainfall in AWM follows a bimodal pattern typical of southern Ghana. The country’s southern region has two distinct rainy seasons, which run from April to June (major rainy season with peak rainfall in June) and September to November (minor rainfall) [33]. CCM covers an area of 122 square kilometers, while AWM spans 447 square kilometers. According to the 2021 Population and Housing Census, CCM has a total population of 189,925 residents (92,790 men and 97,135 women), whereas AWM has 136,882 residents (64,198 men and 72,684 women) [34].

CCM is situated in a secondary forest zone, featuring a 13-kilometer coastline and a warm climate. Rainfall is highest in May, June, and October, with drier periods occurring between November and February. The primary economic activities in CCM revolve around fishing and related industries. In contrast, AWM is predominantly a farming community, with agriculture serving as the backbone of its local economy.

The selection of these two locations was based on their differing ecological and socioeconomic characteristics, which may influence the prevalence and outcomes of malaria and type 2 diabetes mellitus (T2DM). This diversity provides a robust setting for investigating the impact of malaria-T2DM comorbidity on immune responses.

### Recruitment of study participants

A total of 144 participants were recruited for the Swedru study from 1^st^ August, 2021–25^th^ May, 2022, comprising four groups: 40 individuals with type 2 diabetes mellitus (T2DM), 25 individuals with both diabetes and malaria (comorbidity group), 41 individuals with malaria only, and 38 healthy staffs from the Cape Coast Teaching Hospital (CCTH) and Agona Municipal Hospital (ASMH) without malaria or diabetes matched by age and sex as controls after screening. Participants were selected from patients seeking medical care for malaria and diabetes at Cape Coast Teaching Hospital and Agona Swedru Municipal Hospital in the Central Region of Ghana. Additionally, diabetic patients attending routine evaluations at the hospitals’ diabetes clinics were considered for inclusion.

### Inclusion and exclusion criteria

The diagnosis of diabetes was based on fasting plasma glucose levels of ≥126 mg/dL (≥7.0 mmol/L) or a 2-hour postprandial glucose level of >200 mg/dL (≥11.1 mmol/L), following WHO criteria [35]. Malaria diagnosis was determined by a positive rapid diagnostic test (RDT) and confirmed through microscopy.

The study excluded individuals under 40 years of age because Type 2 diabetes prevalence increases substantially after age 40 in our study population, facilitating recruitment of sufficient diabetic participants, longer exposure to malaria parasite as well as reducing chances of recruiting type 1 patients. Also, those who declined to provide informed consent, and individuals diagnosed with type 1 diabetes due to its different pathological pathway or gestational diabetes following screening. Pregnancy status was assessed through self-reporting during the screening interview, and women who reported being pregnant or suspected pregnancy were excluded from the study. Additionally, women of childbearing age with uncertain pregnancy status were offered urine-based pregnancy testing (using standard hCG rapid test kits, DiaSpot HCG Test Strip) before enrollment. Additionally, participants with pre-existing conditions such as fibrosis, hepatitis, HIV, pancreatitis, viral or bacterial infections, rheumatoid arthritis, asthma, and heart failure were excluded to minimize confounding factors.

### Plasmodium parasite antigens

The recombinant antigens used in this study were selected based on their involvement in different stages of the life cycle of the parasite. These antigens included Apical Membrane Antigen 1 (AMA-1), which has 25–545 amino acids (FVO strain), expressed in Pichia pastoris, a methylotrophic yeast (Biomedical Primate Research Centre, Rijswijk, The Netherlands) [36] Glutamate-Rich Protein (GLURP-R0/GLURP-R2) (amino acids 25–514, F32 strain) was expressed in E. coli [36], (Infection-Immunity Department of the Statens, Serum Institute of Copenhagen [Denmark]). Pfs48/45 has a tobacco etch virus (TEV) protease site inserted between R0 and the 6C fragment. The pure, correctly folded Pfs48/45 6C protein was produced with high yield in Drosophila S2 cells [37,38]. PRIMVAC consists of the DBL1x-2x fragment of variant 2 chondroitin sulphate A (VAR2CSA) from the 3D7 strain of *Pf* parasite clone produced in France [39,40]. Circumsporozoite Protein (CSP) is a 44-amino acid NANP repeat-sequence peptide of the circumsporozoite protein of Pf parasite synthesised and Merozoite Surface Protein 1 and 3 (MSP-1,3) by Sygma Genosys [41] These antigens were selected based on their established roles as vaccine candidates in malaria research. GLURP-R2 and GLURP-R0 are both conserved blood-stage antigens that have shown promise in vaccine development; GLURP-R2 contains highly immunogenic epitopes, while GLURP-R0 represents the full N-terminal region and has been associated with protective immunity [42,43]. Both were included to assess differential antibody responses to different regions of the same protein. MSP1 and MSP3 are well-characterized merozoite surface proteins that are primary targets of protective immunity [31]. AMA-1 is essential for erythrocyte invasion and is a leading vaccine candidate [44]. CSP is expressed during the sporozoite stage and is the basis of the RTS,S vaccine [45]

### Ethical considerations

The study received ethical approval from the Ethical Review Committee of Cape Coast Teaching Hospital (CCTHERC/EC/2021/058). The study objectives, procedures, and confidentiality measures were clearly explained to all participants. Written informed consent was obtained before enrollment, ensuring voluntary participation in both data and sample collection.

## Data collection

### Socio-demographic and medical data

Information on socio-demographic characteristics and anthropometric measurements was collected using structured questionnaires. Additionally, participants’ medical records were reviewed to confirm their clinical history and health status.

### Blood sample collection

5 ml of venous blood sample was collected from each participant into a sterile EDTA tube. The sample in the sterile EDTA tube was centrifuged at 1500 rpm for 5 minutes. The plasma obtained was stored in cryovials at −80ºC at the Noguchi Memorial Institute for Medical Research (NMIMR) laboratory until ELISA was performed.

### Diabetes and malaria testing

Fasting blood was collected from participants through finger pricks for malaria and diabetes status using a Rapid Diagnostic Test (RDT) kit (CareStart TM Malaria PfHRP2/pLDH Ag RDT, Access Bio, Inc., USA) and glucometer (*On Call Plus*), respectively. Digital infrared thermometer (JiaLe Technology Co., Ltd.; ET002B) was used to check participants temperature. Fever was defined as ≥37.5°C per WHO guidelines [46] The fasting blood sugar (FBS) tests were performed in duplicates to ensure consistency and accuracy. The results of the tests were interpreted following WHO guidelines [46].

### Parasite density count

About 2 drops of whole blood were used to prepare thick and thin blood smears and stained with 10% Giemsa stain solution for species identification and parasite density count using microscopy [47]. Parasite density was estimated by counting parasites against leukocytes as described elsewhere [47]. Malaria status and parasitaemia (parasites/μL) were confirmed using microscopy by two microscopists (certified medical laboratory scientists with at least 5 years of experience in malaria microscopy) whose results were blinded to each other’s finding. Varying results between the two microscopists repeated and confirmed by a third microscopist. All positive samples were identified as *P. falciparum*, with no other Plasmodium species detected in this study

### Measurement of antibody levels against malaria recombinant antigens

Indirect ELISA was used to evaluate the levels of IgG against these antigens (GLURP-R2, GLURP-RO, MSP3, MSP1, AMA-1 and CSP). Briefly, the 96-well microtiter plates (Thermo Fisher Scientific, Rochester, NY, USA) were coated with the recombinant antigens at a concentration of 1.00 μg/well and incubated overnight. Plates were washed three times with 200 μL/well of wash buffer (PBS with 0.05% Tween-20), blocked with 200 μL/well of blocking buffer (3% BSA in PBS-Tween 20), and incubated at room temperature for 1 hour. The plates were then washed thrice. The plasma samples (1:200 dilution) were diluted in antibody buffer (washing buffer with 1% skimmed milk) and added to the plates (100 μL/well). Plates were incubated for 1 hour at room temperature in an orbital shaker to avoid non-specific binding. Afterwards, plates were again washed three times, and the goat anti-human IgG was diluted 1:3000 (v/v) in antibody buffer according to the manufacturer’s recommendations, and 100 μL/well was added to the plate. After 1 hour incubation at room temperature, the plates were washed five times for 10 min and incubated with 100 μL of TMB substrate solution for 30 min at room temperature, in the dark. Finally, 100 μL/well of stop solution (0.2M H_2_SO_4_) was added to stop the reaction. The absorbance optical density (OD) was obtained at 450 nm and 530 nm with a reference of 620 nm in a microplate reader (Bio-Rad Model 680 Microplate Reader). ODs were converted into arbitrary units using the ADAMSEL software.

### Statistical analysis

SPSS (Version: 20.0) software was used to analyze the research data. Kolmogorov Smirnov test was used to test for normality. Continuous variables were represented as mean and standard deviation, whilst categorical variables were presented as frequencies and percentages. Variables that do not follow a normal distribution were represented by median and interquartile range and compared using the Mann-Whitney U test. Categorized data were analyzed using the Chi-square test. Arbitrary antibody units were transformed into Log10 units. Multivariable linear regression analysis was used to evaluate the relationship between infection status and antibody levels, adjusting for age and sex as confounders in malaria immunity to assess the independent effect of comorbidity status on antibody levels. Linear regression was used to evaluate the relationship between age and antibody levels. *P* < 0.05 was considered to be statistically significant.

## Results

### Demographic and clinical characteristics of the study participant

The study examined four groups, namely diabetes only (N = 40), Co-Morbidity (N = 25), Malaria Only (N = 41), and Negative Control (N = 38), representing a total of 144 study participants.

The mean ages across the groups ranged from 57.2 ± 10.3 to 58.3 ± 13.0 years, with no significant difference observed (p = 0.971). Gender distribution showed a marked predominance of female participants, 115 (79.86%), across the groups compared to their male counterparts, 27 (20.14%). A significant difference between the groups was seen in the temperature readings (p = 0.001). Mean temperatures varied from 36.3°C ± 0.7 to 37.0°C ± 0.8, with the Malaria Only group showing the highest mean temperature (37.0 ± 0.8°C) (Table 1).

**Table 1:** Demographic and clinical Characteristics of the Study participant.

Variables	Diabetes Only (N = 40)	Co-Morbidity (N = 25)	Malaria Only (N = 41)	Negative Control (N = 38)	P-value
**Age (Mean±SD)**	57.2 ± 10.5	57.2 ± 12.7	58.3 ± 13.0	57.4 ± 10.3	0.971^**a**^
**Gender**					
*Male*	12(41.4)	5 (17.2)	5(17.2)	7 (24.1)	0.243^**b**^
*Female*	28 (23.9)	20 (18.8)	36 (30.8)	31(26.5)	
**Marital Status**					
Widowed	9(25.7)	4(11.4)	12(34.3)	10(28.6)	0.377^a^
Single	0(0)	2(50)	1(50)	1(50)	
Married	21(25.39)	17(20.5)	24(28.9)	21(25.3)	
Divorced	10(45.5)	2(9.1)	4(18.2)	6(27.3)	
**Temperature (Mean±SD)**	36.4 ± 0.8	36.5 ± 0.8	37.0 ± 0.8	36.3 ± 0.7	**0.001** ^**a**^

^a^One-way ANOVA; ^b^Chi-square test of association; Values in bracket are percentages.

### Parasitaemia level among malaria patients with or without diabetes

The study showed a significantly higher parasitaemia level 1702 (IQR1 = 926.50, IQR3 = 4102) among the malaria-diabetes comorbidity group in contrast to the malaria only group 932 (IQR1 = 722.50, IQR3 = 1321), (p = 0.02) as presented in Table 2.

**Table 2:** Parasitaemia level among malaria patients with or without diabetes.

Infection Status	N	Median (IQR1, IQR3)	P value
Malaria +Diabetes	25	1702(926.50,4102)	0.02
Malaria Only	41	932(722.50, 1321)	

N= frequency, IQR1= Lower Interquartile Range, IQR3= Upper Interquartile Range, Chi-square test of association. Unit of parasitaemia = parasite/ µL

### Antibody response against malaria vaccine candidate antigens among study groups

Antibody levels against GLURP-R2 were significantly 1.60-fold higher among the comorbidity group (β = 0.47 [95%CI = 0.10, 0.83], p = 0.01) while Malaria Only group constituted a significantly 1.43-fold higher (β = 0.36 [95%CI = 0.04, 0.69], p = 0.03) compared to the negative control group while adjusting for age and sex. However, among the comorbidity groups only, IgG levels against GLURP-R0 were 1.43-fold higher (β = 0.36 [95%CI = 0.03, 0.68], p = 0.03) while MSP1 recorded 1.42-fold higher (β = 0.35 [0.09, 0.61], p < 0.05) compared to the negative control group. Conversely, we observed no significant differences in the IgG response to MSP3, AMA1, and CSP among the different groups (p > 0.05) (Table 3).

**Table 3:** Association between IgG response and Infection status against malaria vaccine candidate antigens.

	Diabetes Only		Comorbidity		Malaria Only	
Antigens	β (95% CI)	P-value	β (95% CI)	P-value	β (95% CI)	P-value
GLURP-R2	0.01 (−0.31, 0.33)	0.95	**0.47 (0.10, 0.83)**	**0.01**	**0.36 (0.04, 0.69)**	**0.03**
MSP3	0.04 (−0.23, 0.31)	0.77	0.11 (−0.20, 0.42)	0.48	0.24 (−0.03, 0.51)	0.08
AMA1	−0.05 (−0.34, 0.23)	0.71	0.02 (−0.31, 0.35)	0.90	0.07 (−0.21, 0.36)	0.61
GLURP-R0	−0.12 (−0.41, 0.17)	0.43	**0.36 (0.03, 0.68)**	**0.03**	0.09 (−0.19, 0.38)	0.52
MSP1	0.05 (−0.18, 0.28)	0.64	**0.35 (0.09, 0.61)**	**<0.05**	0.16 (−0.06, 0.39)	0.16
CSP	−0.08 (−0.27, 0.10)	0.37	0.03 (−0.18, 0.23)	0.80	0.03 (−0.15, 0.22)	0.73

Note: Multivariate regression analysis adjusting for age and sex. β, estimated effect of covariate on antibody levels; CI, confidence interval; Arbitrary antibody units were log_10_ transformed; Co-morbidity (malaria and Diabetes). Negative control group was set as reference for the model.

Further multivariate regression analysis adjusting for age and sex showed that comorbidity individuals had significantly 1.38-fold higher IgG levels against GLURP-R0 1.38-fold higher (β = 0.32 [95%CI = 0.07, 0.59], p = 0.027) while against MSP1 recorded 1.34-fold higher (β = 0.29 [95%CI = 0.02, 0.47], p = 0.048) compared with the malaria-only infected group. However, there was no significant difference in IgG levels against GLURP-R2, MSP3, AMA1 and CSP between the co-morbid and the malaria-only group (p > 0.05) (Table 4)

**Table 4:** Antibody responses against malaria antigens among comorbidity groups relative to malaria infection.

	Comorbidity	
Malaria antigens	β (95% CI)	P-value
GLURP-R2	0.13 (−0.23, 0.48)	0.478
MSP3	−0.12 (−0.39, 0.16)	0.405
AMA1	−0.05 (−0.33, 0.24)	0.741
GLURP-R0	0.32 (0.07, 0.59)	**0.027**
MSP1	0.29 (0.02, 0.47)	**0.048**
CSP	0.004 (−0.22, 0.23)	0.975

Note: Multivariate regression analysis adjusting for age and sex. β, estimated effect of covariate on antibody levels; CI, confidence interval; Arbitrary antibody units were log_10_ transformed; Co-infected (malaria and Diabetes). Malaria only group was set as the reference for the model.

**Fig 1:**
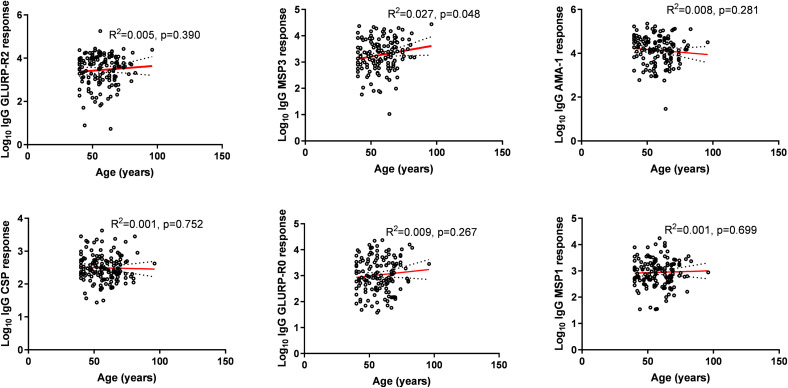
Relationship between age and antibody responses to malaria vaccine candidate antigens.

While MSP-3 demonstrated a weak but statistically significant positive correlation with age (p = 0.048), GLURP-R2, MSP1, AMA1, GLURP-R0, and CSP do not exhibit clear linear trends with age (p > 0.05. The scatter plots have varying degrees of data dispersion, with some antigens (e.g., GLURP-R2, MSP1) showing tighter clustering compared to others (Fig 1).

## Discussion

The increasing prevalence of metabolic disorders, including type 2 diabetes mellitus (T2DM), in developing regions has become a major public health concern [48,49]. This is particularly evident in sub-Saharan Africa, where the co-occurrence of communicable and non-communicable diseases presents a unique challenge to healthcare systems [49]. Given the evolving landscape of disease burden, comorbidity research is gaining prominence in global health discussions [19]. This study examined the impact of T2DM and malaria co-morbidity on IgG responses against malaria vaccine candidate antigens, offering valuable insights into how diabetes may alter immune responses to malaria infection. Our study focused specifically on *Plasmodium falciparum*, the most predominant and clinically significant species globally [32].

A key finding of this study was the significantly higher parasitaemia in individuals with both diabetes and malaria compared to those with malaria alone **(**1702, IQR = 926.50–4102 vs 932, IQR = 722.50–1321, p = 0.02**).** Similar observations have been reported by Abdulai et al., where T2DM-malaria comorbidity exhibited greater parasitemia than those with malaria alone [50]. This phenomenon is attributed to metabolic changes in diabetic individuals that enhance *Plasmodium falciparum* virulence. Hyperglycemia, elevated glycated hemoglobin, and insulin resistance have been linked to increased parasite proliferation [19]. Also, hyperglycemia facilitates malaria parasite growth by enhancing glucose uptake in infected red blood cells, resulting in higher parasitemia in diabetic patients [51]. Additionally, diabetes-related immune dysfunction, such as impaired macrophage activity and compromised neutrophil function, may contribute to reduced parasite clearance and increased parasitemia [52].

At the cellular level, insulin signaling in red blood cells (RBCs) phosphorylates cytoskeletal proteins and enhances glucose uptake, creating a favorable environment for parasite multiplication [19,51]. As seen in vitro, increased glucose availability may support *P. falciparum* development by giving the parasite easily accessible resources for rapid growth [18]. Research has shown that higher fasting blood glucose (FBG) encourages the development of parasites, leading to higher parasite density and malaria severity [17]. These findings suggest that hyperglycemia not only increases susceptibility to malaria but may also exacerbate its severity, leading to worse clinical outcomes. Given the growing prevalence of diabetes in malaria-endemic regions, further research is needed to explore how metabolic disorders modulate malaria pathogenesis.

Our results demonstrated that individuals with diabetes-malaria co-morbidity exhibited significantly higher IgG levels against GLURP-R2*,* GLURP-R0*,* and MSP1 compared to the control group. Notably, IgG levels against GLURP-R2 were elevated in both the malaria-only and comorbidity groups, indicating that malaria infection alone can enhance immune recognition of this antigen [53]. However, the stronger response observed in co-morbidity individuals suggests that diabetes may modulate immune responses in a manner that enhances antibody production [54]. These findings align with previous studies highlighting GLURP-R2 as a highly immunogenic malaria antigen that elicits robust antibody responses [55,56]. The enhanced IgG response in co-morbidity individuals may be due to prolonged or repeated exposure to *P. falciparum* antigens, coupled with diabetes-associated immune dysfunction. Notably, research from Myanmar has shown that cytophilic IgG1 and IgG3 antibodies against GLURP-R0 *and* MSP3 are associated with protection against clinical malaria [57]. The complementary action between MSP3*- and* GLURP- specific IgG3 antibodies further supports the potential benefit of incorporating these antigens into malaria vaccine formulations.

After adjusting for age and sex, multivariate regression analysis confirmed that individuals with diabetes-malaria co-morbidity had significantly higher IgG levels against GLURP-R0 and MSP1 compared to the malaria-only group. This is consistent with previous findings demonstrating that co-infections can enhance immune responses to malaria antigens. For instance, co-infection with *Necator americanus* and *P. falciparum* has been associated with increased IgG responses to the *GMZ2* malaria vaccine candidate [58]. The full-length MSP protein (MSP1FL) in particular is one of the most significant targets of functional antibodies that support malaria-preventive immunity [59]. Increased anti-MSP1 responses have clinical significance, as evidenced by recent studies showing that vaccination with MSP1FL produces long-lasting IgG antibody titers that surpass those seen in semi-immune individuals from Africa [59].

Recent research has demonstrated that increased levels of IgG to GLURP, together with MSP1 and MSP3, are linked to a lower incidence of malaria, supporting the significance of the improved response to GLURP-R0 [60]. Accordingly, the improved GLURP-R0 responses seen in our diabetic group should offer some protection against malaria, possibly counteracting some of the higher risk of severe malaria that is generally linked to diabetes.

The increased antibody responses seen in diabetics who also have malaria could be the result of multiple interrelated processes. First, type 2 diabetes’s chronic inflammatory state, which is characterized by high levels of pro-inflammatory cytokines like TNF-α, IL-6, and IL-1β, may lead to an environment of increased immune activation that, ironically, promotes the production of antigen-specific antibodies [61]. When B cells and plasma cells come into contact with malaria antigens, this long-term low-grade inflammation may act as a biological adjuvant, preparing them for stronger antibody responses.

Moreover, insulin resistance and hyperglycemia, two metabolic abnormalities associated with diabetes, may change the metabolism of immune cells in ways that promote the generation of antibodies [62]. According to recent studies, the availability of glucose can affect B cell activation and antibody release, with some metabolic conditions possibly favoring more robust humoral immune responses [62,63]. While IgG levels against MSP-3 exhibited positive correlations with age, no clear linear trends were observed for *GLURP-R2, MSP-1 AMA1*, *GLURP-R0*, or *CSP*. Prior studies have reported that antibody responses to key malaria antigens, including *GLURP*, *MSP3*, and *AMA1*, increase with age and are associated with reduced malaria incidence [42,55]. This age-dependent increase in protective antibodies has been linked to the gradual acquisition of immunity through repeated exposure to malaria parasites [64]. People who live in regions where malaria is prevalent usually gradually gain immunity via frequent exposure. IgG antibodies, particularly subclasses IgG1 and IgG3, which are directed against malaria vaccine candidate antigens including GLURP, MSP1, and AMA1, play a major role in mediating this protection [53]. Therefore, rather than an acute reaction or diabetes-specific modulation alone, the increased IgG responses seen in both the malaria-only and comorbid (malaria + diabetes) groups may be partly due to cumulative exposure. Additionally, antibodies that prevent *P. falciparum* adhesion to endothelial receptors have been shown to increase with age, further contributing to protective immunity [65].

These findings from this study, highlights critical considerations for malaria vaccination in populations with rising diabetes rates. For vaccine development, while immunogenicity may be preserved or boosted in diabetic populations, efficacy could be diminished if diabetes affects antibody functionality [66]. This suggests the need for additional booster doses or adjuvants to improve antibody quality in diabetic individuals in malaria-endemic areas. Clinical trials should stratify participants by metabolic disease status to evaluate differential vaccine efficacy.

The strengths of our study lie in the fact that it discusses the immunological relationship between type 2 diabetes and malaria in an endemic setting, an issue that has received little attention but is becoming more crucial; Well-defined comparison groups were included into the research design to enable the evaluation of antibody responses in various illness situations and to quantify antibodies, we used standardized ELISA procedures with quality controls, thus, reducing potential biases. However, the authors acknowledge that our inability to target specific haemoglobin variants, nutritional status, small sample size, absence of avidity test and absence of PCR and HbA1c results are major limitations of this study.

## Conclusion

Our findings suggest that diabetes-malaria co-morbidity enhances *P. falciparum* parasite proliferation and alters antibody responses to malaria vaccine candidate antigens. The significantly higher IgG levels observed in co-infected individuals highlight the potential impact of diabetes on malaria immunity. Understanding the mechanisms underlying these immune interactions is critical for developing effective malaria vaccines, particularly in populations with a high burden of metabolic disorders. Future research should explore how diabetes-associated immune dysfunction influences long-term malaria immunity and vaccine efficacy. Also, future studies should incorporate PCR and assess clinical symptoms more comprehensively.

## Supporting information

S1 Data Malaria Diabetes Comorbidity study data. (XLSX)
